# SynthSR-generated 3D T1-weighted MRI from routine 2D clinical images: Validation for VSRAD analysis

**DOI:** 10.3389/fneur.2025.1645891

**Published:** 2025-10-16

**Authors:** Tsukasa Koike, Akio Morita, Tetsuro Sekine, Tetsuya Sakai, Takahiro Tsuchiya, Atsumi Takenobu, Akira Teraoka

**Affiliations:** ^1^Department of Neurosurgery, Teraoka Memorial Hospital, Hiroshima, Japan; ^2^Teraoka Geriatric Healthcare Center, Teraoka Memorial Hospital, Hiroshima, Japan; ^3^Tokyo Rosai Hospital, Japan Organization of Occupational Health and Safety, Tokyo, Japan; ^4^Department of Radiology, Nippon Medical School Hospital, Tokyo, Japan; ^5^Department of Computer Science and Engineering, Waseda University, Tokyo, Japan

**Keywords:** 3D Slicer, Alzheimer disease, FreeSurfer, Mild cognitive impairment, MRI, SynthSR, Voxel-Based Morphometry, VSRAD

## Abstract

**Background:**

The Voxel-based Specific Regional Analysis System for Alzheimer’s Disease (VSRAD), a voxel-based morphometry tool quantifying medial temporal lobe atrophy as region-specific *Z*-scores, is widely used in clinical practice for detection of Alzheimer’s disease (AD). However, it typically require high-resolution 3D T1-weighted MRI, which is often difficult to acquire in elderly or cognitively impaired patients. This study aimed to evaluate whether 3D volumes generated by SynthSR from 2D T1-weighted MRI can yield volumetric and VSRAD-derived indices that are comparable to those from standard 3D images, by assessing agreement, rank consistency, and diagnostic performance.

**Methods:**

In this retrospective single-center study, MRI data from 75 patients were analyzed using both standard 3D T1-weighted images and SynthSR-generated 3D volumes reconstructed from 2D T1-weighted sequences. Regional brain volumes and four key Z-score indices from VSRAD were compared using Wilcoxon signed-rank tests with Bonferroni correction, robust Bland–Altman analysis, Spearman’s rank correlation, and receiver operating characteristic (ROC) curve analysis focusing on Score 1 “Severity.”

**Results:**

All Z-score indices and segmented volumes showed significant absolute differences between the two methods (*p* < 0.0071), with SynthSR-based data generally yielding larger volume estimates. Despite these differences, Spearman’s *ρ* remained consistently high (*ρ* > 0.7) for brain volume and Score 3 “Ratio,” and other clinically relevant indices also demonstrated moderate correlations. ROC analysis demonstrated high value of the area under the curve (AUC) values for both standard 3D volumes (0.90) and SynthSR-generated 3D volumes (0.96), with no statistically significant difference between the two methods (*Z* = 0.009, *p* = 0.99, DeLong’s test).

**Conclusion:**

Although SynthSR-based images produced systematically different absolute values, they preserved rank-order correlations and maintained diagnostic performance comparable to that of standard 3D volumes in VSRAD analysis. Considering that conventional 3D acquisitions are often difficult to obtain in elderly patients undergoing dementia screening, SynthSR-based reconstruction may represent a practical alternative in routine clinical practice, particularly for Score 1 “Severity,” the most clinically relevant marker of hippocampal atrophy.

## Introduction

According to the World Health Organization, the global population aged over 60 is projected to reach 2.1 billion by 2050, while the number of individuals over 80 is expected to triple to 426 million between 2020 and 2050 (1). Dementia is a prevalent condition, with its global incidence steadily increasing in recent years (2, 3). Timely diagnosis of conditions such as Alzheimer’s disease (AD) and mild cognitive impairment (MCI) is crucial for maintaining patients’ quality of life, as early intervention can potentially slow disease progression (4–6). Hippocampal atrophy is a characteristic feature of AD, making the recognition of this atrophy at an early stage particularly useful (7).

One commonly used image analysis tool is VSRAD (Voxel-based Specific Regional Analysis System for Alzheimer’s Disease), developed by Eisai, a computer-aided diagnostic system designed to support theclinical diagnosis of AD at an early stage (8, 9). VSRAD uses modified voxel-based morphometry (VBM) software. This software combines Statistical Parametric Mapping 8 (SPM8) and Diffeomorphic Anatomical Registration Through Exponentiated Lie Algebra (DARTEL) algorithm for the detection and quantitative assessment of AD to compare a patient’s brain MRI to a normative database of healthy individuals (10). Thus, VSRAD primarily evaluates atrophy in the medial temporal lobe (including the hippocampus and parahippocampal gyrus), visualizing results as Z-score maps and quantifying the degree of atrophy in various brain regions with high versatility (11, 12). VSRAD is used in over 3,000 facilities in Japan and has been validated across multiple centers. This corresponds to roughly 30–40% of all MRI-equipped facilities nationwide. VSRAD ordinarily requires three-dimensional T1-weighted volume images, which take about five minutes to acquire. However, acquiring such data is often challenging in patients with suspected dementia due to poor compliance and motion artifacts, which frequently degrade image quality. Consequently, routine clinical imaging still relies mainly on two-dimensional sequences. However, the relatively large slice thickness of typical 2D scans (approximately 4–5 mm) poses a particular challenge for quantitative analyses like VBM, especially when analyzing historical 2D image data.

SynthSR is an image super-resolution technique recently implemented in FreeSurfer, a comprehensive open-source software package used for processing and analyzing brain MRI images. It has been shown to be capable of generating high-quality, high-resolution images from low-resolution MRI scans, which ultimately improves the accuracy of brain structure analysis (13, 14). Compared to conventional interpolation methods, SynthSR has been reported to reproduce more detailed brain structures and can complement older, low-resolution MRI datasets (15). Furthermore, SynthSR has served as a benchmark for newer models such as LoHiResGAN, which convert low-field into high-field equivalents (16), and has been integrated into workflows for Alzheimer’s disease assessment using hippocampal and white matter hyperintensity quantification (17). Recent studies have also applied SynthSR to ensure anatomical consistency in youth cohorts (18) and to generate cerebrospinal fluid (CSF) volumetrics predictive of stroke outcomes (19). These applications highlight SynthSR’s potential in both standardizing heterogeneous datasets and expanding the utility of legacy MRI data or data from resource-limited settings.

In this study, we evaluated the agreement and comparability of VSRAD analysis results between 3D volumes generated from 2D T1-weighted images and standard 3D T1-weighted volumes, with particular focus on between-method agreement, rank-based consistency, and diagnostic performance. We assessed whether SynthSR-generated 3D images from 2D inputs could yield results comparable to standard 3D images, potentially offering a more practical approach for dementia assessment.

## Materials and methods

### Study design

This single-center, retrospective, observational study was conducted using data collected from November 2021 to January 2022. The study cohort consisted entirely of patients who underwent head MRI because of suspected cognitive decline in routine clinical practice. VSRAD analysis was performed on both conventional 2D T1-weighted images and standard 3D T1-weighted images. Given that the objective of this study was methodological rather than diagnostic, the analysis focused exclusively on this clinically relevant elderly cohort, without introducing a separate healthy control group or stratification by dementia subtype. This design allowed us to directly assess the comparability between standard 3D and SynthSR-generated 3D volumes under real-world clinical conditions.

### Image acquisition

The head MRI was performed during a routine clinical examination. A 1.5 Tesla MRI system (SIGNA Explorer 1.5 T, GE Healthcare Japan, Tokyo, Japan) with 8-channel coil was used. 2D T1-weighted images (T1w_2D) were acquired using periodically rotated overlapping parallel lines with enhanced reconstruction (PROPELLER). The imaging parameters were as follows: the matrix size was 224 × 224, slice thickness was 6.00 mm, repetition time (TR) was 567 ms, echo time (TE) was 12 ms, flip angle was 90°, field of view (FOV) was 24.0 cm. Acquisition time was 105 s. 3D T1-weighted images (T1w_3D) were acquired using spoiled gradient recalled acquisition in steady state (SPGR) in the sagittal plane. Its parameters: the matrix size was 256 × 256, slice thickness was 1.50 mm, TR was 11.4 ms, TE was 4.48 ms, flip angle was 15°, FOV was 25.6 cm. Acquisition time was 232 s. T1w_3D was used for control data. For each patient, both 3D and 2D T1-weighted images were acquired on the same day during a single MRI session.

### Generating 3D volume data

**Figure 1:**
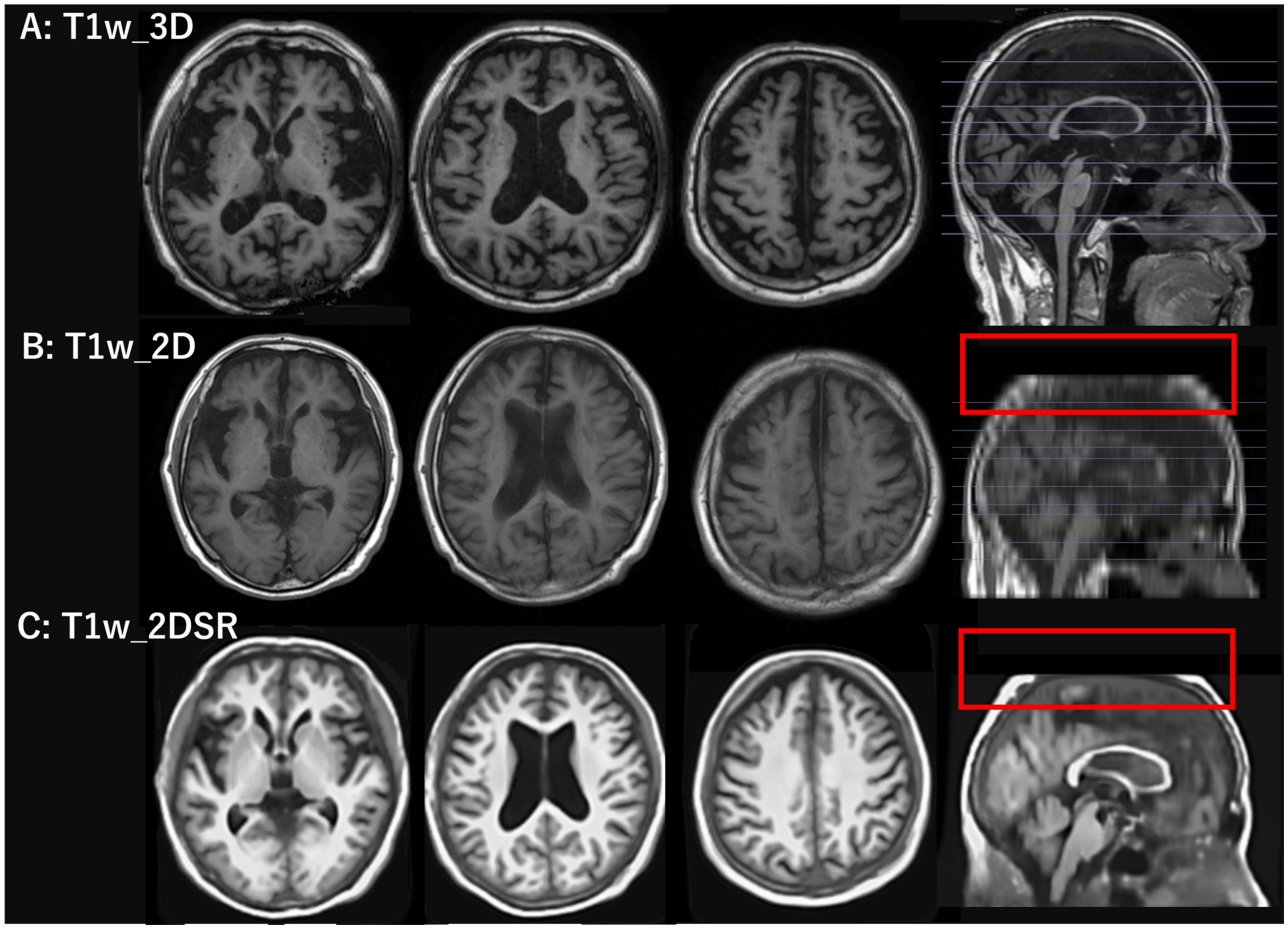
T1-weighted images used in the standard and proposed methods. The top row shows 3D T1-weighted images acquired using spoiled gradient recalled acquisition in steady state (SPGR) sequence with the standard method. The middle row shows 2D T1-weighted images acquired using periodically rotated overlapping parallel lines (PROPELLER), which are commonly used in clinical settings. The bottom row shows 3D volume data generated from 2D T1-weighted images using FreeSurfer’s SynthSR tool. The red rectangles in the middle and bottom rows indicate that the parietal region was not included in the original scan and therefore could not be generated.

T1w_2D images were converted from DICOM to NIfTI format using MRIcroGL (20). The 3D volume data (T1w_2DSR) were generated using FreeSurfer’s SynthSR from T1w_2D modified to NIfTI file format (13–15). SynthSR was used with default settings, except that 4 CPU threads were specified. The 3D volume was output as a 1.0 mm MPRAGE-like image with standard contrast, bias magnetic field correction, and inpainting of white matter lesions. The average time for T1w_2DSR generation was 77 s. The generated T1w_2DSR was in NIfTI format. Since VSRAD required DICOM in the sagittal orientation, the image was reoriented from axial to sagittal and converted to DICOM format using 3D Slicer (Figure 1) (21). All processing was performed under Windows Subsystem for Linux 2 (WSL2) using a laptop computer ThinkPad X1 Extreme (Lenovo Japan LLC, Tokyo, Japan) with the following specifications: Intel Core i7-8750H CPU (up to 4.10 GHz), 32 GB RAM (Intel Corp., California, USA), and NVIDIA GeForce GTX 1050 Ti with Max-Q Design (Nvidia Corp., California, USA).

### VSRAD advance analysis

**Figure 2:**
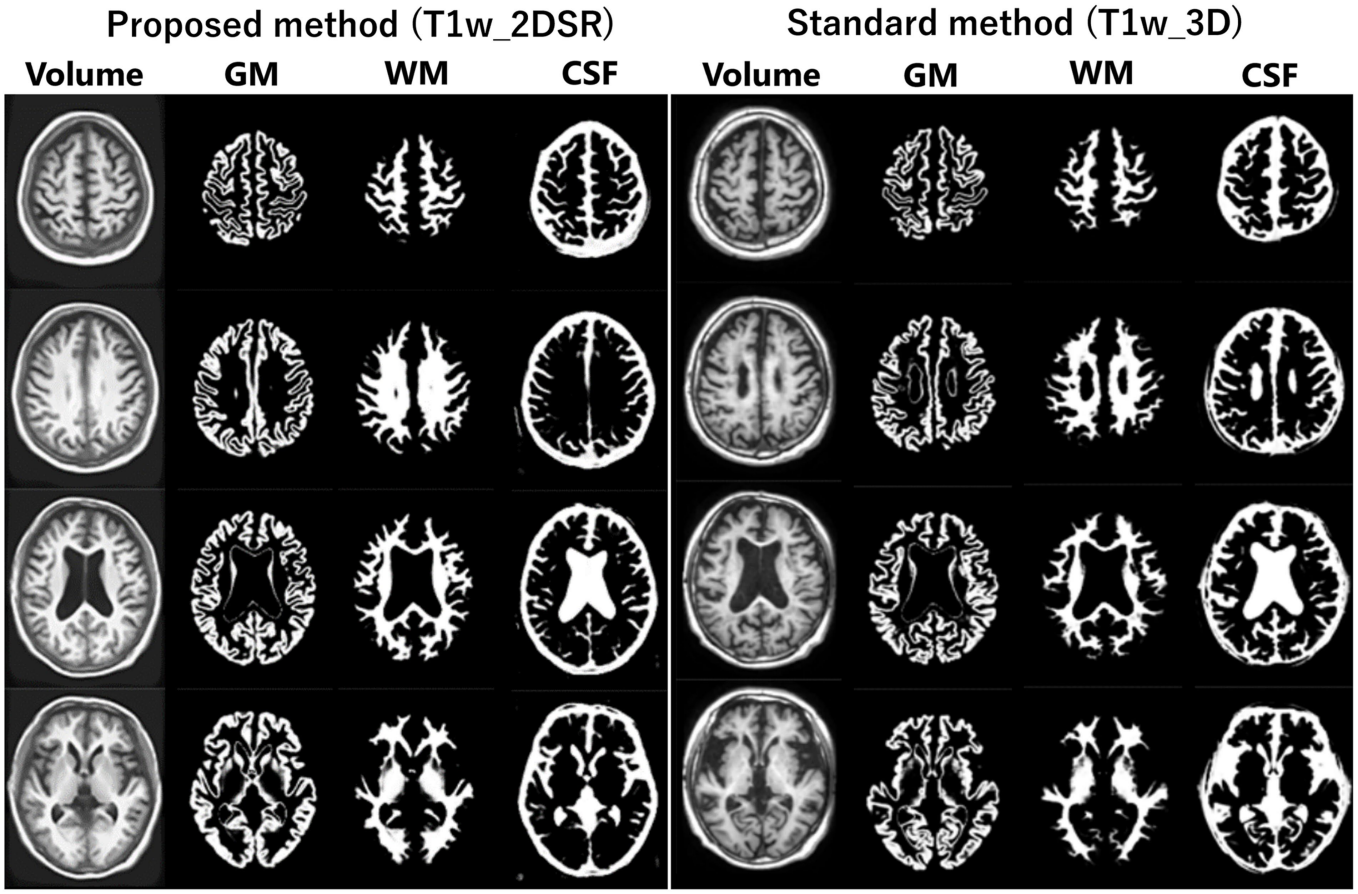
Segmented images obtained by VSRAD. The left panel shows the VSRAD analysis results using the SynthSR-based method (T1w_2DSR). Segmented gray matter (GM), white matter (WM), cerebrospinal fluid (CSF) images were successfully generated. The right panel shows the comparison of segmented GM, WM and CSF images using the standard method (T1w_3D). These results indicate that the volume data generated by SynthSR demonstrated comparable segmentation performance to 3D data from standard methods.

Several versions of VSRAD have been developed. In this study, we used “VSRAD advance,” which was based on SPM 8 and incorporated DARTEL (11, 22, 23). VBM was performed on both T1w_2DSR and T1w_3D, yielding data on segmented white matter (WM), gray matter (GM), CSF (Figure 2). Four Z-scores reflecting the degree of atrophy in the specific volume of interest (VOI) were automatically calculated and provided by VSRAD advance, based on comparing each patient’s data with an internal database of 80 healthy volunteers. The *Z*-score was defined as [(control mean) – (individual value)]/(control standard deviation) (11). The *Z*-scores generated by VSRAD advance visualize and quantitatively evaluate the degree of gray matter atrophy in the regions of interest, primarily the parahippocampal gyrus including the medial temporal lobe, which is closely associated with Alzheimer’s disease, by measuring the degree of deviation from the normative brain database. The four scores were as follows: Score 1 “Severity”: Z-score reflecting the severity of GM atrophy in the VOI. Score 2 “Extent”: the extent of GM atrophy in the VOI. Score 3 “Ratio”: the ratio of the extent of GM atrophy in the VOI to the whole brain. Score 4 “Maximum”: the maximum z-score of the severity of GM atrophy in the VOI of AD (24–28). To mitigate volumetric inaccuracies introduced by non-linear spatial normalization and Gaussian smoothing in the DARTEL pipeline, intermediate WM and GM segmentation files were used for estimating native-space volumes. Total brain volume was calculated as the sum of the WM and GM volumes (WM + GM). Measurements were performed using the Segment Statistics module of 3D Slicer (13).

### Statistical analysis

In this study, to compare the standard method and the proposed method, statistical analyses were performed using R (version 4.3.3; R Foundation for Statistical Computing, Vienna, Austria) and RStudio (2023.06.1; Posit, Boston, USA) (29, 30). The normality of the data distribution for each variable was assessed using the Shapiro–Wilk test. Variables that did not follow a normal distribution (*p* < 0.05) were analyzed using non-parametric methods (31). We conducted a Wilcoxon signed-rank test (paired, two-sided) to determine if there were statistically significant differences between the T1w_3D and T1w_2DSR groups (32). Bonferroni correction (significance threshold at *α* = 0.05/7 ≈ 0.0071) was performed to compare several indices simultaneously (33). Given the non-normal distribution of differences and the presence of outliers, we next implemented a robust Bland–Altman plot using the median bias and interquartile range (IQR), which provides a more reliable interpretation of agreement by reducing the influence of skewness and extreme values on the summary estimates (34–36). We plotted the differences and means of the two methods and calculated the 95% confidence interval of the differences. Spearman’s rank correlation analysis was employed to evaluate monotonic relationships between the volume estimates derived from the standard method and the proposed method. This non-parametric approach was appropriate given the lack of normality and the interest in rank-based consistency. The correlation strength was interpreted as follows: *ρ* < 0.3 = weak, 0.3–0.7 = moderate, and > 0.7 = strong correlation (37). Receiver operating characteristic (ROC) curve analyses were conducted to assess the diagnostic performance between patients diagnosed AD and cognitively normal controls. The final diagnoses were determined by dementia specialists based on clinical, neuroimaging, and neuropsychological information. The area under the curve (AUC) was calculated for both methods (38, 39), and optimal cutoff points were identified via the Youden Index (40). ROC curves were compared using DeLong’s test implemented in the pROC package in R (41).

### Ethical considerations

The study was reviewed by the ethics committee of our institution (FY2023-02). An opt-out notice was published on the institution’s website.

## Results

**Figure 3:**
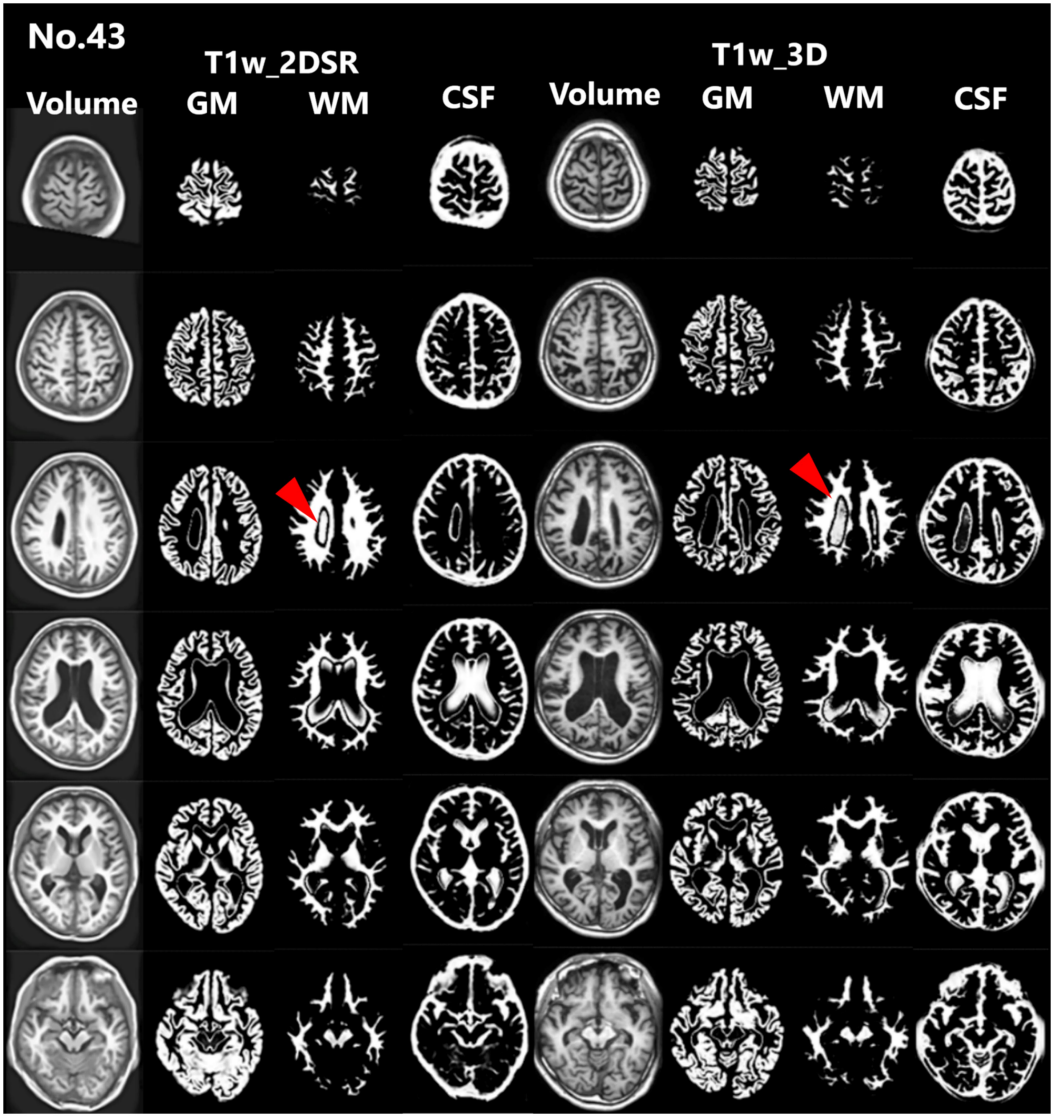
Representative excluded cases of atrophy. In Case no. 43, severe brain atrophy led to segmentation failure in both the standard and proposed methods, with portions of the ventricles erroneously classified as white matter (red arrowheads).

**Figure 4:**
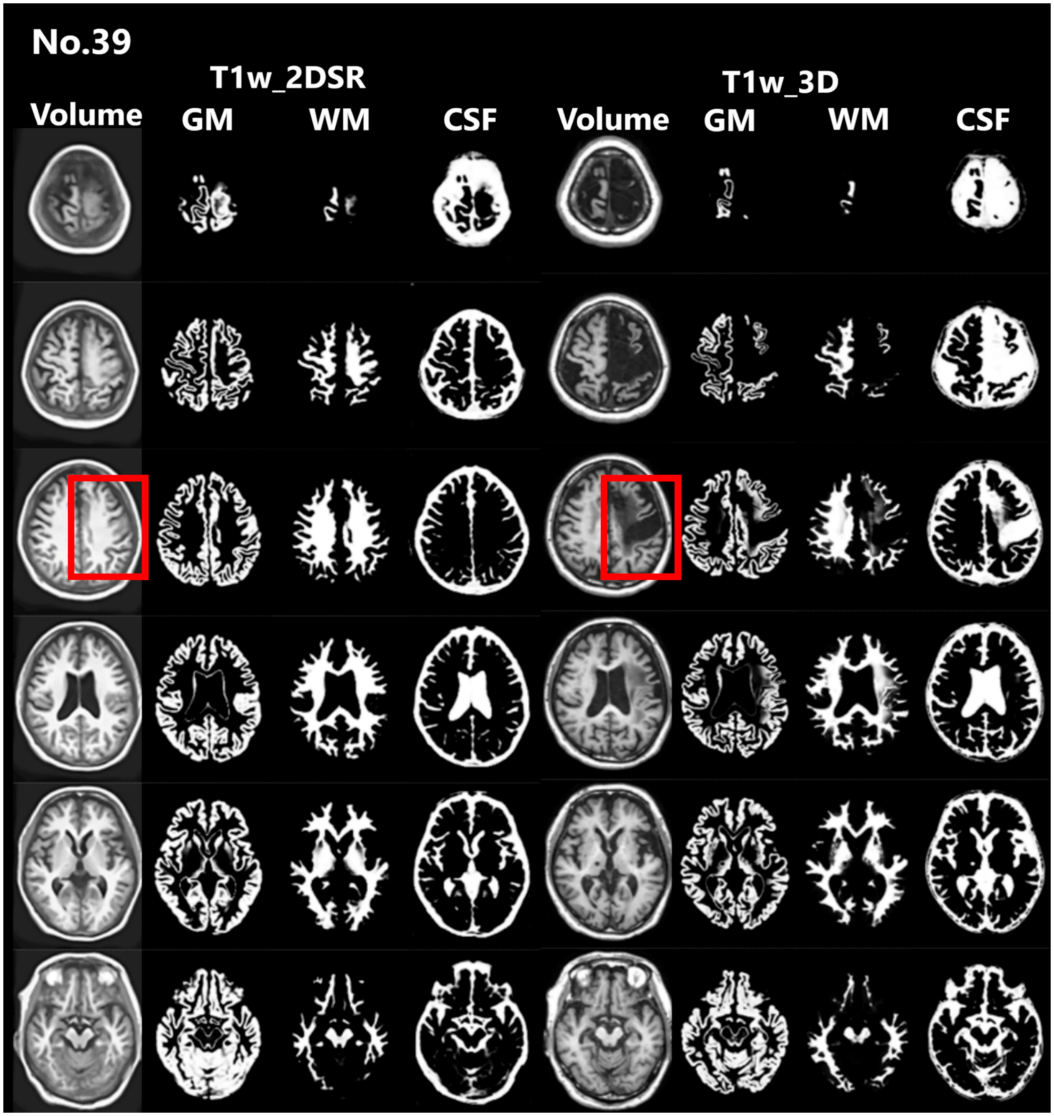
Representative excluded cases of stroke. Case no. 39 shows a patient with structural brain lesions due to stroke. In the 3D volume data acquired using the standard method, a low-signal area was observed in the left frontal lobe, consistent with cerebral infarction. In contrast, in the 3D volume generated by SynthSR, the lesion area appeared artificially filled in (red rectangles).

Seventy-five patients underwent MRI during the study period. Of these, 21 (28.0%) were male. The mean age was 83.5 years (range, 61–107 years). This cohort reflects the typical population undergoing dementia screening in Japan. For all cases, 3D volume data (T1w_2DSR) were successfully generated from T1w_2D using SynthSR. The generated T1w_2DSR did not reconstruct the parietal CSF region because it was not imaged in the original T1w_2D. Each output image was independently reviewed by two neurosurgeons. Cases with obvious motion artifacts and disrupted WM and GM segmentation in the control T1w_3D images were excluded from the analysis. Among the excluded cases, four (Cases 22, 26, 43 and 63) had showed segmentation errors involving the ventricles and GM due to brain atrophy (Figure 3), five (Cases 7, 11, 12, 69 and 72) showed segmentation failure caused by motion artifacts; and two (Cases 15 and 39) had structural brain lesions due to stroke (Figure 4). A total of 64 cases were analyzed after excluding 11 cases. After VSRAD analysis, four scores and three volumes were calculated. For these evaluated indices, normality was not met in most cases (Shapiro–Wilk test, *p* < 0.05), and thus non-parametric comparisons were adopted.

Wilcoxon signed-rank tests were conducted across seven indices. As shown in Table 1, all comparisons showed statistically significant differences between the two methods. The proposed method tended to yield larger volume measurements in most VSRAD scores and segmented volumes. After applying Bonferroni correction for multiple comparisons (*n* = 7), all *p*-values remained below the significance threshold of 0.05, confirming the robustness of the findings.

**Table 1:** Comparison of seven indices between the standard and proposed methods using the Wilcoxon signed-rank test.

Variable	Standard median (Q1–Q3)	Proposed median (Q1–Q3)	Raw *p* value	Bonferroni-adjusted *p* value
Score 1 “Severity”	1.23 (0.80–1.75)	2.06 (1.22–3.03)	1.59 × 10^−8^	1.43 × 10^−7^
Score 2 “Extent”	5.44 (4.74–6.31)	6.91 (4.62–9.64)	7.27 × 10^−5^	6.54 × 10^−4^
Score 3 “Ratio”	11.72 (1.81–35.67)	38.87 (9.65–75.85)	2.77 × 10^−9^	2.49 × 10^−8^
Score 4 “Maximum”	2.11 (0.37–5.55)	5.18 (1.71–7.82)	4.76 × 10^−6^	4.28 × 10^−5^
Volume of GM	482.24 (435.84–537.23)	516.33 (449.53–566.62)	2.08 × 10^−6^	1.87 × 10^−5^
Volume of WM	555.18 (518.74–582.94)	578.31 (540.41–602.12)	1.49 × 10^−5^	1.34 × 10^−4^
Volume of Brain	1061.69 (973.23–1112.59)	1111.66 (1026.41–1173.98)	5.09 × 10^−7^	4.58 × 10^−6^

*All *p*-values are obtained using the Wilcoxon signed-rank tests.

Bonferroni correction was applied to control for family-wise error rate (*α* = 0.05, *n* = 7).

GM, gray matter; WM, white matter.

**Figure 5:**
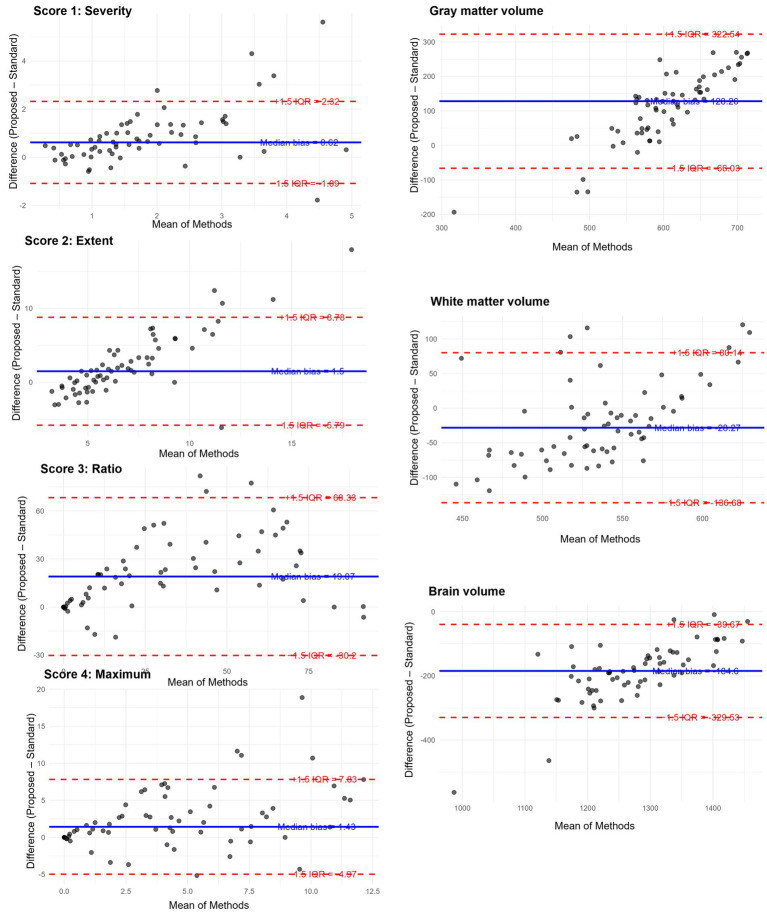
Robust Bland–Altman plots for the results of VSRAD analysis. Each plot illustrates the difference between the proposed method (T1w_2DSR) and the standard method (T1w_3D), based on VSRAD-derived volume measurements. The differences are plotted against the median of the two methods. The solid blue line indicates the median difference (bias), and dashed red lines represent the robust 95% limits of agreement, defined as the median ± 1.5 × IQR. Outliers beyond the boundaries are marked and quantified. IQR: interquartile range.

Robust Bland–Altman analysis was conducted across seven indices. For each measurement pair, the median of the difference (Proposed method – Standard method) and the IQR were used to estimate robust limits of agreement defined as median ± 1.5 × IQR (Table 2). Among all indices, IQRs and derived limits of agreement varied across metrics, reflecting higher variability in volume-based measures than in *Z*-scores. Outlier analysis revealed that fewer than 10% of cases fell outside the robust limits of agreement for all variables, with the highest outlier proportions observed in gray matter volume and Score 1, both at 9.4%. Notably, score 3 “Ratio” and WM showed the largest absolute median differences, suggesting consistent deviations between the two methods (Figure 5).

**Table 2:** Results of Robust Bland–Altman analysis comparing VSRAD scores and volumetric measurements between the standard and proposed methods.

Variable	Median difference	IQR	Lower limit	Upper limit	Outlier count	Outlier percent
Score 1 “Severity”	0.62	1.14	−1.09	2.32	6	9.4
Score 2 “Extent”	1.50	4.86	−5.79	8.78	5	7.8
Score 3 “Ratio”	19.07	32.84	−30.20	68.33	3	4.7
Score 4 “Maximum”	1.43	4.27	−4.97	7.83	5	7.8
Volume of GM	−28.27	72.27	−136.68	80.14	6	9.4
Volume of WM	128.26	129.53	−66.03	322.54	4	6.2
Volume of Brain	−184.60	96.62	−329.53	−39.67	5	7.8

Each row presents the median difference (Pro − Std), the interquartile range (IQR), and the corresponding robust limits of agreement (LoA), calculated as median ± 1.5 × IQR. The number and percentage of outliers beyond the robust LoA are also reported.

GM, gray matter; WM, white matter.

**Figure 6:**
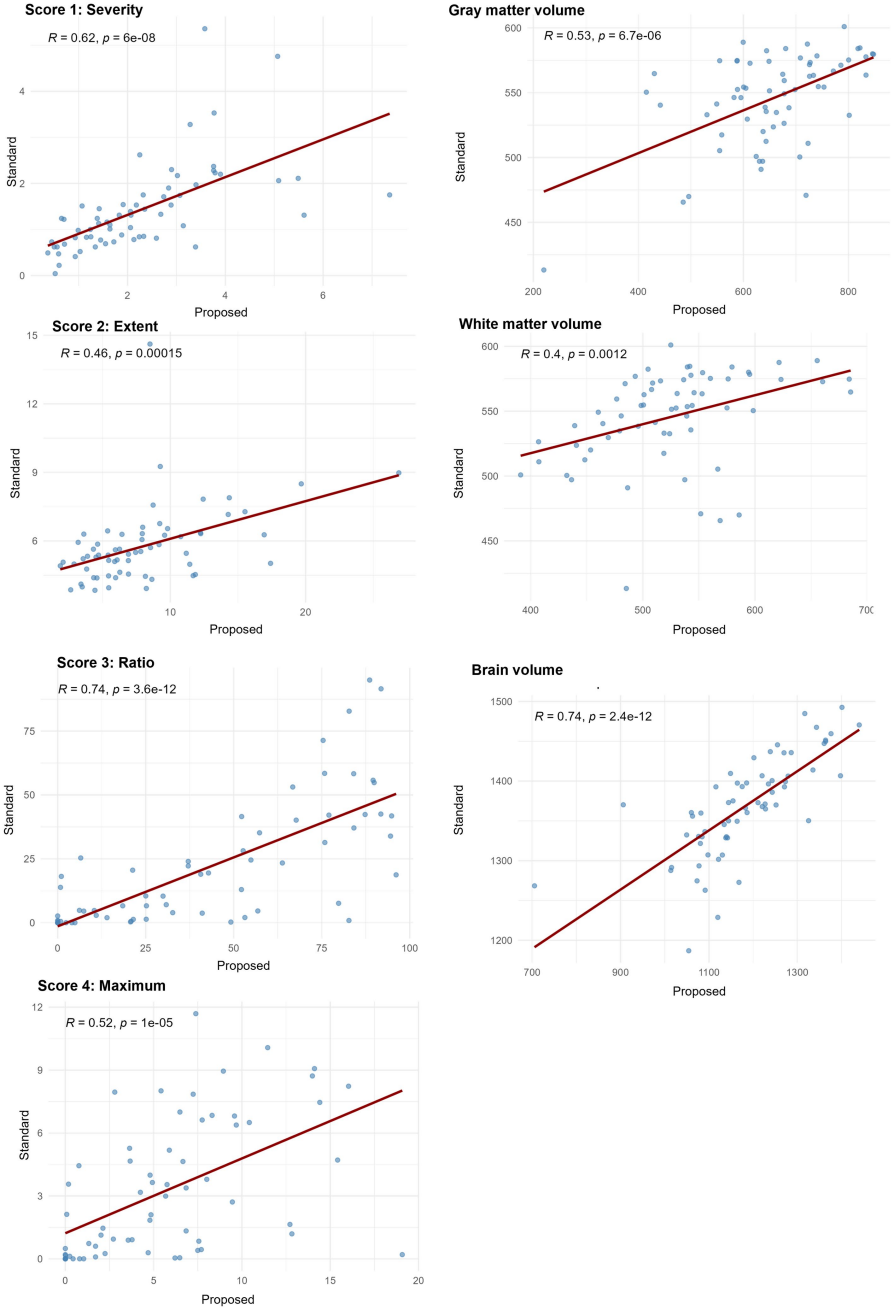
Spearman’s correlation coefficients between VSRAD analysis and volumetric measurements from the standard method and the proposed method. Scatter plots illustrate the rank-order association between measurements obtained from the standard method with 3D T1-weighted images (T1w_3D) and SynthSR-generated 3D volumes from 2D T1-weighted images (T1w_2DSR). Spearman’s correlation coefficients (*ρ*) and corresponding *p*-values are displayed in each plot. Correlation strength was interpreted as follows: ρ < 0.3 = weak, 0.3–0.7 = moderate, and > 0.7 = strong correlation. Score 3 “Ratio” and brain volume showed strong correlations and the other indices showed moderate correlations.

Spearman’s correlation coefficients between the standard and proposed methods across all seven indices ranged from 0.40 to 0.74, with all values indicating statistically significant positive correlations (*p* < 0.001). Strong correlations (*ρ* > 0.7) were observed in score 3 “Ratio” and brain volume, while the remaining indices showed moderate correlation (0.3 < *ρ* < 0.7), indicating consistent ranking between the two methods (Figure 6).

**Figure 7:**
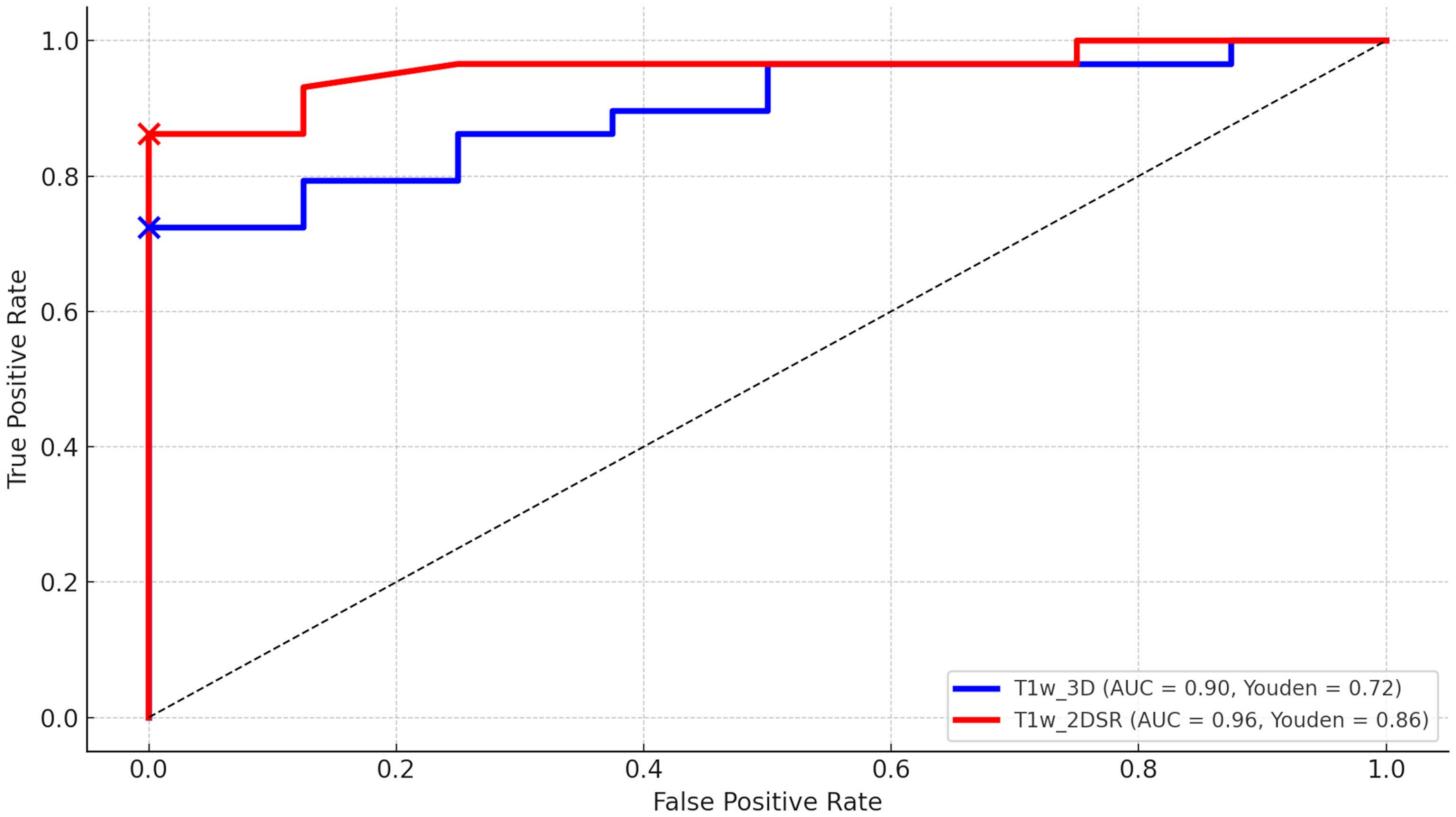
The receiver operating characteristic (ROC) curves of VSRAD Score 1 “Severity.” The ROC curve, area under the curve (AUC), and Youden index were compared between the standard method (T1w_3D) and the proposed method (T1w_2DSR). The standard method showed an AUC of 0.90 and a Youden index of 0.72, while the proposed method showed an AUC of 0.96 and a Youden index of 0.86.

Twenty-nine patients were diagnosed with AD, 8 were considered cognitively normal, and the rest were diagnosed with other dementias. ROC curves were drawn for AD and cognitively normal controls based on Score 1 “Severity” in VSRAD analysis. For the standard method (T1w_3D), AUC = 0.90, Youden Index = 0.72, and for the proposed method (T1w_2DSR), AUC = 0.96, Youden Index = 0.84 (Figure 7). DeLong’s test revealed no statistically significant difference between the AUCs of the standard and proposed methods (*Z* = 0.009, *p* = 0.993), with a 95% confidence interval of −13.85 to 13.97. These results indicate that the diagnostic performance was comparable between the two methods.

## Discussion

This study compared four VSRAD scores and three volumetric measurements derived from the standard 3D T1-weighted MRI (T1w_3D) and the proposed SynthSR-based method (T1w_2DSR) using a multipronged statistical approach. The Wilcoxon signed-rank test revealed statistically significant differences across all four VSRAD scores and the three segmented brain volumes. The proposed method generally yielded larger volumetric estimates, even after Bonferroni correction. These differences indicate systematic biases, likely attributable to the SynthSR algorithm. Robust Bland–Altman analysis provided further insight by showing small median differences in most scores and volumes (30–32). These discrepancies may reflect the generative behavior of SynthSR. SynthSR likely compensated for thinned gray matter in cases of severe parenchymal atrophy, thereby increasing the apparent volume in the T1w_2DSR images. This effect was particularly evident in cases with ischemic lesions or cortical atrophy, where segmentation of GM, WM, and CSF failed. These cases were characterized by severe brain atrophy due to aging and enlarged ventricles (Figure 3) (42, 43). Furthermore, GM and WM volumes were larger in T1w_2DSR than in T1w_3D. The overall stronger degree of atrophy may be attributed to the advanced age of our cohort (mean age 83.5 years), compared to the VSRAD reference data cohort, which consisted of healthy subjects aged 54–86 years (44, 45).

Because Spearman’s *ρ* captures monotonic associations, several metrics such as Score 3 “Ratio” and the total brain volume demonstrated strong correlations, underscoring the reliability of SynthSR. Other indices showed moderate correlations. Our findings are generally consistent with the previous validation studies of SynthSR, particularly regarding correlation strength (46–48). Iglesias et al. demonstrated compatibility with morphometric properties derived from FreeSurfer and reported minimal bias across various brain structures (14). From a technical perspective, the use of Spearman’s rank correlation and robust agreement analysis follows recent recommendations for comprehensive evaluation of segmentation pipelines (49, 50).

The ROC curve analysis demonstrated high diagnostic accuracy for both T1w_3D (AUC = 0.90, Youden Index = 0.72) and T1w_2DSR (AUC = 0.96, Youden Index = 0.86). These findings suggest that both methods effectively differentiate between AD and normal cases. Although the proposed method (T1w_2DSR) showed numerically higher diagnostic metrics, this difference was not statistically significant (DeLong test: *Z* = 0.009, *p* = 0.99). Importantly, in clinical practice, Score 1 “Severity”—which reflects the degree of gray matter atrophy in the medial temporal lobe—is a critical index for early AD detection and monitoring. The consistently high AUC and favorable diagnostic characteristics of the proposed method support its use as a reliable alternative when standard 3D imaging is not feasible. Notably, SynthSR has been employed in low-field MRI settings (15, 17) and has also been used for segmentation correction in lesioned or incomplete scans (50, 51). These prior studies, along with applications in predicting thrombectomy outcomes (19) and tracking cortical lesions in traumatic brain injury (52), support the utility of SynthSR-enhanced reconstructions not only for advanced neuroimaging workflows but also for routine clinical scenarios, especially when standard 3D acquisitions are unavailable or degraded.

In DARTEL-based analyses such as VSRAD, the MRI data quality is critical, as factors such as head motion can significantly influence the results (53–56). In the present study, the quality of image data acquisition likely affected the results. Various strategies have been proposed to overcome challenges in acquiring 3D T1-weighted images. For example, Katayama et al. suggested using scout images for positioning to shorten scan time, although they reported significant differences in gray matter volume (55, 57, 58).

We suggest that SynthSR-enhanced volumetry is a viable alternative to standard 3D acquisitions in both clinical and research settings, particularly when scan time constraints or motion artifacts are of concern. In our cohort, T1w_2DSR reduced scan time to less than half (105 vs. 232 s). This reduction likely helped mitigate motion artifacts. Nevertheless, five T1w_3D cases had to be excluded due to motion artifacts. It is worth noting that our cohort was older than the typical target population for VSRAD, and the absence of parietal regions in the 2D images precluded intracranial volume comparisons. While this limitation restricts direct volume ratio analyses, Z-score-based assessments, such as those used in VSRAD, may still benefit from SynthSR-derived inputs. Moreover, this approach may also be applicable for longitudinal monitoring of individual patients. Previous studies have reported that SynthSR can improve the quality of low-field or heterogeneous MRI datasets, and it has been applied for segmentation correction in lesioned brains, harmonization across scanner types, and enhancement of legacy datasets (15, 17, 19). However, to our knowledge, its direct application to generate 3D-equivalent volumes from 2D T1-weighted inputs for VSRAD analysis in Alzheimer’s disease has not been systematically evaluated. The innovative aspect of this study lies in leveraging paired 2D and 3D acquisitions obtained on the same day to validate the feasibility of SynthSR-based reconstructions specifically for VSRAD indices, which are widely used in clinical practice in Japan. This approach demonstrates that retrospective 2D scans can be repurposed for quantitative dementia assessment, potentially expanding access to VSRAD analysis in settings where 3D scans are unavailable or degraded.

The greatest strength of this study lies in its comprehensive and robust statistical evaluation framework. However, several limitations must also be acknowledged. These include the lack of manual segmentation as ground truth, the generally older age of subjects, and the use of single-center data. Another important limitation of this study is that our cohort consisted predominantly of elderly individuals. This reflects the real-world demographics of patients undergoing dementia screening in Japan, where most individuals referred for MRI are already in advanced age. While this population is clinically relevant, the advanced age makes it challenging to fully disentangle age-related brain atrophy from Alzheimer’s disease–related neurodegeneration. Validation in younger subjects within the typical age range of AD onset would therefore be desirable. However, such a dataset was not available for the present retrospective single-center study. Future multicenter investigations that include younger cohorts and a broader age distribution will be essential to confirm the generalizability of our findings. Detailed clinical or biomarker characterization was not systematically available and was not the primary aim of this methodological validation study. Our primary aim was to assess comparability between standard and SynthSR-derived 3D volumes, independent of clinical diagnosis. Future studies should include multicenter validation across diverse populations, along with manual labeling to establish a reliable reference standard. Furthermore, although SynthSR revealed significant differences in many indices, the correlation coefficients remained relatively strong. This suggests that further optimization is warranted for specific brain structures and pathological conditions. Notably, the retrospective use of archival 2D images enabled by SynthSR may facilitate large-scale longitudinal studies. This approach holds promise for uncovering novel insights into neurodegeneration and its potentially modifiable risk factors.

## Conclusion

This study focused on methodological validation rather than on diagnostic accuracy. Specifically, we evaluated whether 3D volumes generated using SynthSR from conventional 2D inputs yielded results comparable to standard 3D acquisition in a clinically relevant cohort of elderly individuals undergoing MRI for suspected cognitive decline. Considering that conventional 3D images are often difficult to obtain in this population, our findings suggest that SynthSR-based reconstruction may represent a practical alternative for VSRAD analysis in daily clinical practice.

## Data Availability

The MRI data have been fully anonymized to protect patient confidentiality, and data will be shared upon reasonable request for research purposes.
